# Competence to Consent and Its Relationship With Cognitive Function in Patients With Schizophrenia

**DOI:** 10.3389/fpsyt.2019.00195

**Published:** 2019-04-12

**Authors:** Norio Sugawara, Norio Yasui-Furukori, Tomiki Sumiyoshi

**Affiliations:** ^1^Department of Clinical Epidemiology, Translational Medical Center, National Center of Neurology and Psychiatry, Kodaira, Japan; ^2^Department of Neuropsychiatry, Hirosaki University School of Medicine, Hirosaki, Japan

**Keywords:** competence to consent, cognitive function, schizophrenia, MacArthur Competence Assessment Tools, decisional capacity

## Abstract

Decisional capacity to consent is an emerging ethical and legal concept, and is closely related to self-determination of patients facing important medical decisions or research participations. Recently, the MacArthur Competence Assessment Tool (MacCAT), a semi-structured interview consisting of four dimensions (Understanding, Appreciation, Reasoning, and Expression of a Choice), was developed to assess the decisional capacity. Decision-making capacity in a group of patients with schizophrenia, as measured by the MacCAT, has been shown to be impaired in comparison with healthy control people. However, this does not necessarily mean the presence of impaired decisional capacity in all cases. Considering the real-world practice of obtaining informed consent from patients with schizophrenia, it is important to evaluate the relationship between psychopathological features and decisional capacity of the illness. Negative symptoms of schizophrenia have been demonstrated to be related to the ability to understand information relevant to the decision, reason rationally, and appreciate a situation and its consequences. On the other hand, positive symptoms, such as delusions and hallucinations have been an inconsistent correlate of poor capacity. Furthermore, some studies indicate that impairment of cognitive function, a core symptom of schizophrenia, could be more largely associated with decisional capacity than positive and negative symptoms. Therefore, it is reasonable to assume cognitive enhancement would enlarge the capacity to consent and promote autonomy in medical treatment and research participation in patients with schizophrenia. Further studies are warranted to elucidate this and related issues.

## Introduction

Competence to consent for individuals with psychiatric symptoms or impaired cognitive functioning has become central to the debate on the informed consent in clinical care and research settings. Clinicians and researchers bear the responsibilities to protect two aspects of human rights; the right of competent patients to make choices about their medical care and the right of incompetent patients to be protected from the potential harm of their decisions (1). However, in real-world clinical settings, some patients with capacity were detained in hospital by law, or other patients with incapacity were admitted to hospital on a voluntary basis (2). In terms of clinical research, some patients with incapacity might have participated in clinical trials with their own consent.

**Figure 1:**
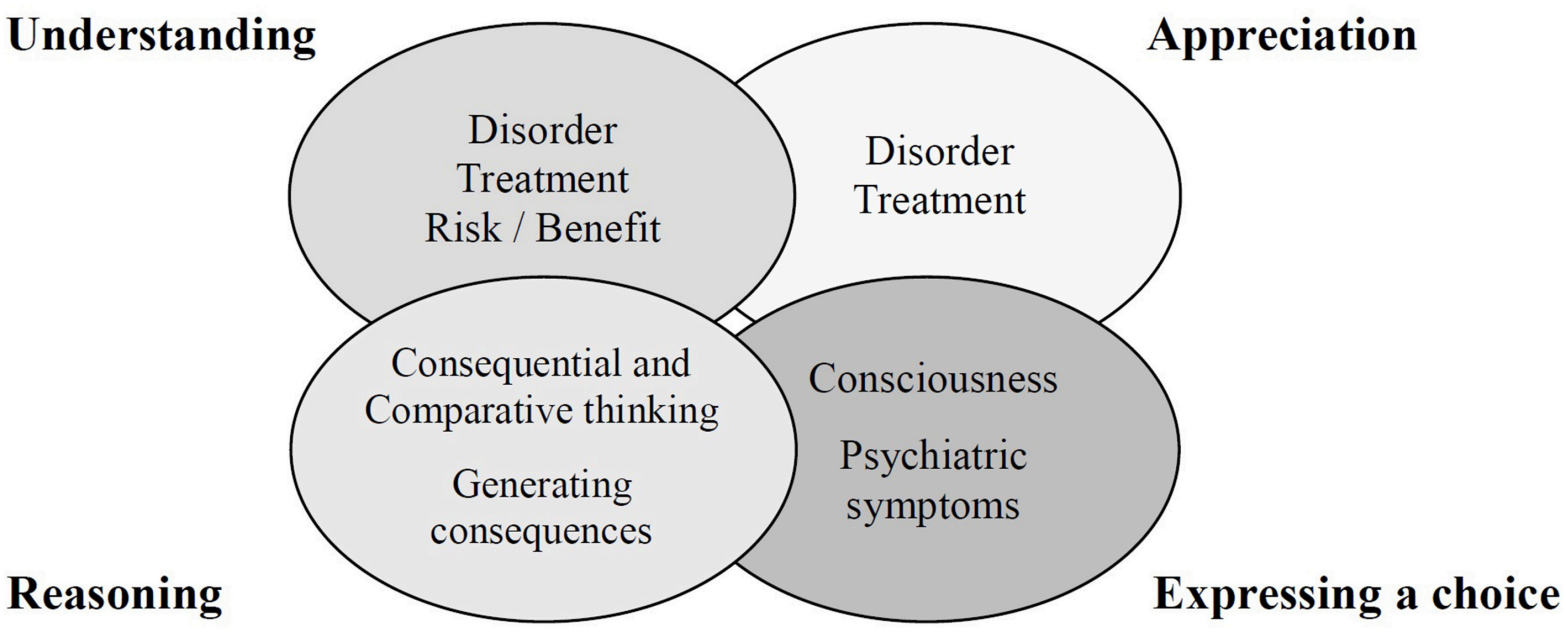
Key elements of the MacArthur Competence Assessment Tool.

Although several decision-making tasks specifically assess decisional capacities (3, 4), the medical/psychiatric literature has commonly cited the following abilities as relevant to capacity for informed consent: (1) understanding information relevant to treatment decision making; (2) appreciating the personal significance of treatment information, especially concerning one's own illness and the probable consequences of one's treatment options; (3) reasoning with relevant information to engage in a logical process of weighting treatment options; and (4) expressing a choice (5). These are also the key elements of the MacArthur Competence Assessment Tool (MacCAT) (6) (Figure 1), which has been widely used for competence assessment (7). However, the MacCAT is not clearly designed to provide a total score for the assessment of decision-making capacity. Furthermore, the abilities assessed in the MacCAT do not necessarily equate to the abilities relevant to the assessment of decision-making capacity in many jurisdictions.

Schizophrenia is a severe mental disorder that generally appears in late adolescence or early adulthood. Epidemiological data indicates that prevalence of schizophrenia is approximately 1% in the worldwide population. Symptoms of schizophrenia are clinically divided into three main categories of positive symptoms (delusions, disordered thoughts, and hallucinations), negative symptoms (restricted affect and drive), and impairments in cognitive function (8, 9). When schizophrenia was first identified by Kraepelin, he noted the fundamental role of cognitive impairment in this disorder, and called this dementia praecox (10). Although patients with schizophrenia are more likely to lack the competence to consent than control groups (6, 11), the diagnosis of schizophrenia cannot be equated with decisional incapacity (12). Many researchers have investigated the associations of competence to consent with positive and negative symptoms of schizophrenia (13–15). Although poor capacity has correlated with negative symptoms more consistently than positive symptoms, high levels of positive symptoms, including disorganization, may affect competence to consent. Furthermore, recent empirical data suggest that neurocognitive functioning could explain a larger proportion of the variance in competence to consent than positive and negative symptoms of schizophrenia (14, 16, 17).

Therefore, the aim of this narrative review is to elucidate (1) the relationships between cognitive function and competence to consent, and (2) the interventions to compensate the decision-making capacity in patients with schizophrenia.

## Cognitive Measures of Multiple Domains and Competence to Consent

Several studies investigated the association of competence to consent and cognitive measures such as the Mini-Mental State Examination (MMSE) (15, 17–19) and the Repeatable Battery for the Assessment of Neuropsychological Status (RBANS) (13, 16). On the whole, poor understanding subscales of MacCAT have been a more consistent correlate of poor cognitive functioning than have other subscales. However, all of above mentioned studies employed MacCAT-Clinical Research (MacCAT-CR) for assessing participants' decision-making abilities for clinical research. So, the potential range of understanding subscales being at least three times those of the other subscales might affect inconsistent results of other subscales.

A longitudinal assessment for capacity, in terms of understanding, was conducted among participants in the Clinical Antipsychotic Trials of Intervention Effectiveness (CATIE) schizophrenia study (20). Over 18 months treatment, poorer baseline neurocognitive composite scores consisting of processing speed, verbal memory, vigilance, reasoning, and working memory, predicted falling below the critical decision-making capacity threshold. In the same analysis, lower baseline scores of understanding subscale also were associated with falling below the threshold during follow-up period.

## Cognitive Measures of Each Domain and Competence to Consent

Verbal communication plays an important role in informed consent (21). Two studies demonstrated the relationship between understanding subscales and verbal cognitive functioning based on the Wechsler Adult Intelligence Scale (WAIS)-Revised (WAIS-R) (22, 23). In addition, other studies showed that appreciation and reasoning subscales are associated with verbal comprehension composed of vocabulary, similarities, and information subtests from the WAIS–Third Edition (WAIS-III) (17, 24). Although previous studies indicated that verbal abilities may predict competence to consent, the relationship between specific dimensions of decisional capacity and individual verbal ability areas is still obscure.

Memory is a complex process, consisting of registration, storage, retainment, and retrieval of information (25). Previous studies from US revealed that the RBANS memory index had significant relationships to understanding, appreciation (13) and reasoning subscales (16). Another study from Hong Kong indicated an association of understanding subscales with immediate and delayed logical memory from the Wechsler Memory Scale (WMS) (5). Furthermore, some studies implicated that memory also underlies an aspect of learning outcome (17, 24). Palmer et al. showed the relationship of learning composite scores with three subscales of MacCAT (understanding, appreciation, and reasoning) (24). They also demonstrated that auditory and visual learning abilities could affect the competence to consent (17).

Working memory is a complex and multifaced construct to store and simultaneously manipulate a limited amount of information during short intervals. This capacity facilitates further cognitive processing, such as response selection relevant for a specific context. Working memory comprises two short-term information storage systems, the visuospatial network for visual material and the phonologic loop for verbal-acoustic material (25). In the CATIE schizophrenia trial (14), this cognitive domain was assessed by a computerized test of visuospatial working memory and letter number sequencing test of auditory working memory. Specifically, working memory performance showed considerable bivariate relationships with the understanding, appreciation, and reasoning subscale scores from the MacCAT-CR. Furthermore, similar results were reported in other studies employing the letter number sequencing test (5) and WAIS-III (17). A previous study indicated an association between language comprehension and working memory for sentences (26). Impairment of working memory in patients with schizophrenia might be consequent upon verbal comprehension deficits.

Information processing represents a cognitive process of taking information and encoding it to be understood and recalled when appropriately cued. Processing speed is the rapidity by which information processing occurs (25). Several studies showed bivariate correlation between processing speed composite scores and understanding, appreciation, and reasoning subscales of MacCAT (14, 17, 24), but only the relationship between processing speed composite scores and understanding subscales was replicated in a multiple regression model (14). In patients with schizophrenia, processing speed performance is strongly associated with global cognitive deficits (27). Thus, processing speed may contribute to the relationship between competence to consent and general cognitive performance.

Executive function involves the simultaneous use of information rather than the basic cognitive process, and governs goal-directed behaviors or adaptive responses to complex or novel situations. Generally, executive function is characterized by several complex mental abilities, including unique skills used for expansion, modulation, and implementation of goal-directed activities (25). This domain of cognitive function has been thought to rely on frontal lobe functions (28). A positive correlation has been reported between performance on the Frontal Assessment Battery and total scores of the MacCAT (29) in chronic schizophrenia patients. Specifically, scores on the understanding subscale were most consistently correlated with executive function (5, 24).

Cognitive underpinnings underlying the limited decisional capacity in psychiatric patients remain to be explored (30). One intriguing study (31) showed that performance on a metacognition test was more closely related to decisional capacity compared to executive function. Metacognition focuses on the level of self-confidence of patients in comparison with actual performance, and predicts performance on the MacCAT-T (31).

## Interventions to Compensate the Decision-Making Capacity

Although antipsychotic medication may improve decisional capacities (32, 33), clinicians and researchers should improve the informed consent process to maximize the decision-making capacities of patients with schizophrenia. Providing information repeatedly and discussing presented information with participants may strengthen the competence to consent (16, 34). Furthermore, consent procedures via multimedia may facilitate the understanding to decide on complex or high-risk protocols (35). Naughton et al. conducted a small uncontrolled study to evaluate the effect of metacognitive training (MCT) to improve a person's awareness of cognitive biases and thinking styles on decision-making capacities (36). MCT was found to elicit improvement in understanding and reasoning, but not appreciation abilities of patients. Furthermore, cognitive remediation may improve competence to consent (37), providing ethically adequate care, as well as clinical improvement.

## Limitations

Several limitations of this review should be acknowledged. The principal limitation is the relatively small sample sizes in most studies, mentioned here, on the relationship between cognitive function and competence to consent in patients of schizophrenia. Secondly, for ethical and legal reasons, only subjects who consented to participate in studies were included. Even among individuals who consented to participate, 10% were too agitated to complete the entire assessments (6), suggesting that generalization of previous findings should be considered with caution. Thirdly, the MacCAT does not have a specific cutoff to dichotomize competence vs. incompetence. This may obscure the associations between cognitive functions and decisional capacities (38). Finally, the MacCAT, which is based on not only medical literature but also existing case law and statutes, does not assess the emotional aspect of decisional capacities. Further studies investigating the relationship between cognitive function and decisional capacities, including both comprehensive emotional and legal aspects, are needed.

## Conclusions

Neurocognitive functioning may explain competence to consent more accurately than positive and negative symptoms. Previous results have not indicated differential relationships between specific cognitive ability areas and decision-making capacity. Interventions with multimedia procedure, MCT, etc. likely enhance competence to consent. Cognitive remediation might provide ethically adequate care as well as clinical improvement. Clinicians and researchers are responsible for maximizing decision-making capacities of patients in the informed consent process. Further studies are warranted to elucidate competence to consent and related issues.

## Author Contributions

NS, NY-F, and TS were involved in the study concept, interpretation of manuscript, critical revision of manuscript for intellectual content, literature review, and drafting of the manuscript.

### Conflict of Interest Statement

The authors declare that the research was conducted in the absence of any commercial or financial relationships that could be construed as a potential conflict of interest.
